# Developing rat testicular organoid models for assessing the reproductive toxicity of antidepression drugs *in vitro*: Testicular organoids to assess antidepressants toxicity

**DOI:** 10.3724/abbs.2022164

**Published:** 2022-11-09

**Authors:** Sixian Wu, Xiaoliang Li, Peiyu Li, Tongtong Li, Gelin Huang, Qun Sun, Andras Dinnyés, Lijun Shang, Wenming Xu

**Affiliations:** 1 Departments of Obstetrics & Gynecology Joint Laboratory of Reproductive Medicine SCU-CUHK Key Laboratory of Birth Defects and Related Diseases of Women and Children Ministry of Education West China Second University Hospital Sichuan University Chengdu 610041 China; 2 Reproduction Medical Centre West China Second University Hospital Sichuan University Chengdu 610041 China; 3 Key Laboratory of Bioresources and Eco-environment of the Ministry of Education College of Life Sciences Sichuan University Chengdu 610041 China; 4BioTalentum Ltd. Gödöllő H-2100 Hungary 5.School of Human Sciences London Metropolitan University London N7 8DB UK

With the increasing incidence of depression worldwide, antidepressant medications are commonly used in males of reproductive age for long-term treatment of depression, as well as other disorders [1]. Antidepressants are known to be associated with sexual side effects [2], such as decreased libido and impotence. Their effects on semen parameters and other markers of male fertility have been less thoroughly described, such as sperm motility and fertilization ability. Therefore, it is critical to determine the potential toxic effects of antidepressants on reproductive organs. A recent study systemically determined the effect of different drugs on the telomere-related DNA damage response of germ cells [3]. In addition, another study showed that mirtazapine has less toxic effects than amitriptyline and other drugs [4]. However, these studies used germ cell lines to determine the phenotype, which limits their potential translational value in toxicity studies in humans. Given the widespread and often long-term use of antidepressant medications, there is an urgent need for further data regarding their impact on semen quality and subsequent male fertility.

The testis is a complex multicellular organ responsible for spermatogenesis. Cell‒cell interactions between somatic and germ cells are essential for normal testis function. Only a few clinical indicators can be used to monitor potential testicular toxicity in drug development; therefore, the evaluation is mainly carried out on animals. This implies high costs and has limitations and potential interfering factors. For example, animals may lose weight after taking the drug, and subsequent alterations in neuromuscular function and mood of the animal may affect mating behavior. Furthermore, animal models might have different pharmacokinetic characteristics, and the overall human translational value might be limited. The development of testicular organoids might save at least €6.7 billion and 48.6 million animals [5]. The existing *in vitro* 2-dimensional cellular models, however, cannot reflect the full interaction among different cell types of the testis. Encouragingly, the rapid development of *in vitro* 3-dimensional organoids has provided a more complex modelling of the structure and function of organs, including tissue-specific progenitor and differentiated cells arranged in an organotypic structure resembling the original organ. Established *in vitro* three-dimensional stem cell models of organogenesis have enormous potential for modelling normal development and disease, as a tool for drug testing, and as a framework for therapeutic testing [6]. Therefore, testicular organoids would be an extremely valuable model to assess reproductive toxicity.

Here, we presented an improved testicular organoid model composed of rat testicular cell homogenates to assess the reproductive toxicity of antidepressants, with special emphasis on mimicking the drugs on different aspects of spermatogenesis and uncovering the underlying mechanisms. The two most frequently used antidepressants, amitriptyline and mirtazapine, were chosen to evaluate their reproductive toxicity effects on spermatogenic cells.

**Figure 1:**
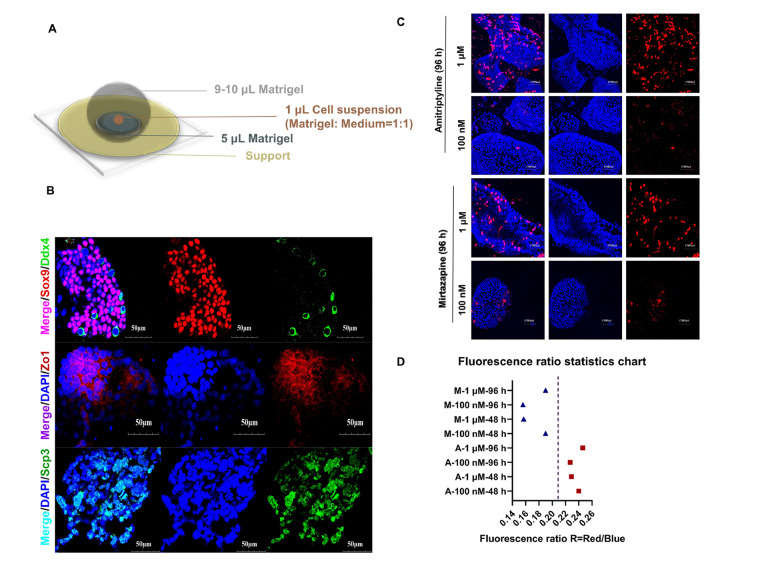
Figure 1 The construction of testicular organoids and the comparison of testicular toxicity between amitriptyline and mirtazapine (A) Testile organoid model. The model has a 3-layer gradient system with careful selection of the cell culture conditions shown in the figure. A layer of 5 μL Matrigel was placed on the support. A 1 μL volume of cell suspension (medium:Matrigel=1:1) was placed on the Matrigel. The outermost layer was wrapped with 9‒10 μL Matrigel. (B) Characterization of organoids. Immunostaining images of the Ddx4 (green), Sox9 (red), Zo1 (red) and Scp3 (green) genes in 7-day cultured organoids. Scale bar= 50 μm. (C) Images of Hoechst (blue) and PI (red) staining after organoids were treated with 1 μM and 100 nM amitriptyline and mirtazapine for 96 h. (D) The fluorescence ratio statistics chart, R=red/blue=PI/Hoechst. The R value was less than 0.21 for the organoids treated with mirtazapine and greater than 0.21 for those organoids treated with amitriptyline.

Testicular organoids are usually cultured by mixing cells and Matrigel directly [7] or by mixing cells with a mixture of medium and Matrigel [8]. We introduced a new 3-layer gradient system (3-LGS) by optimizing the testicular organoid culture conditions, including the number of cells and the concentration of Matrigel. The best results were achieved by using a small volume of Matrigel and a low number of cells (15 μL and 40,000 cells, respectively) in a mixture of medium with a ratio of cells and Matrigel (1:1) between two layers of pure Matrigel ( Figure 1A). These cells from rat testis were digested into a mixture of fragments and cells. Then, all the cells were combined for subpackaging and culture. Nearly 50 organoids can be obtained from one rat testis.

To verify our model, we evaluated spherical–tubular structures composed of Sertoli cells, germ cells, and spermatocytes using the relevant markers SRY-box transcription factor 9 (Sox9), DEAD-box helicase 4 (Ddx4) and synaptonemal complex protein 3 (Scp3), respectively ( Figure 1B). Ddx4 and Scp3 positivity indicate the presence of germ cells, which means that the organoids have the potential to differentiate to initiate spermatogenesis. Sertoli cells are thought to be indispensable supporting cells of spermatogenesis. They act as the main mediator of androgen action in spermatogenesis and are assumed to provide nutrients contributing to the development and maturation of germ cells. The positive staining of Scp3 ( Figure 1B) proved that there are spermatocytes in the organoid. The presence of spermatocytes ( Figure 1B) also indicated that our organoid system could contain germ cells, and the origin of the spermatocytes remains unclear at this stage. Other structural features of this improved testicular organoid model, such as the possible presence of a blood-testis barrier (BTB), were demonstrated by the expression of tight junction protein 1 (Zo-1) ( Figure 1B). Of course, further functional evidence of BTB in the organoid requires further experimental support, such as dye penetration experiments. Ki67 staining showed that the cells were proliferating ( Supplementary Figure S1).

**Figure 2:**
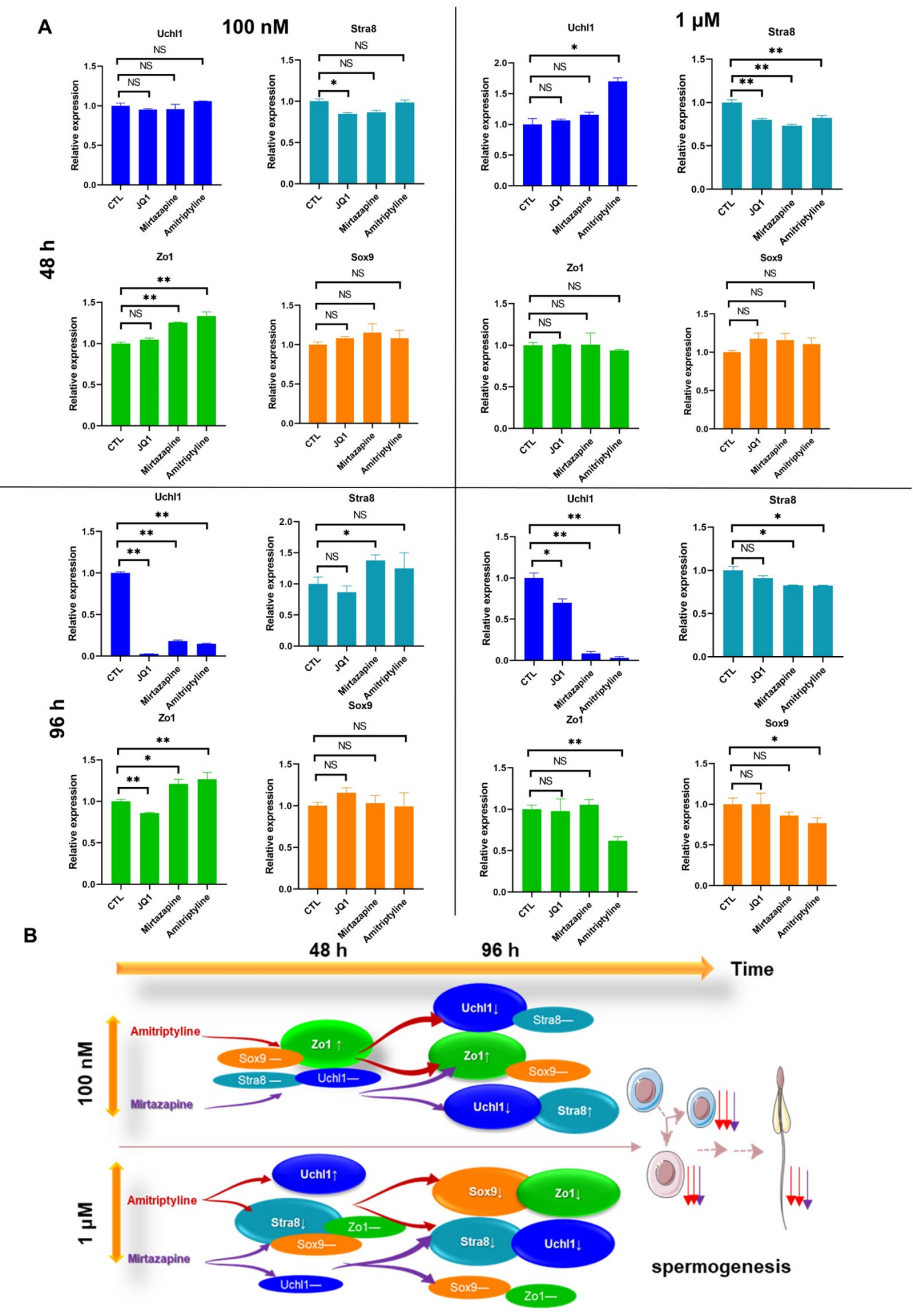
Figure 2 The effects of amitriptyline and mirtazapine on the expressions of key genes related to spermatogenesis (A) The mRNA expression of Zo1, Sox9, Stra8 and Uchl1 after treatment with 1 μM and 100 nM mirtazapine and amitriptyline for 48 and 96 h. JQ1 was used as a positive control. Gapdh was used as an internal reference gene for qPCR. The number of animals in each group n=3, * P<0.05, ** P<0.01. (B) A schematic diagram of the blockade of spermatogenesis with amitriptyline and mirtazapine. “↓” indicates downregulation, “↑” indicates upregulation, “–” indicates no change, “↓ (purple)” indicates the degree to which mirtazapine affects spermatogenesis, “↓ (red)” indicates the degree to which amitriptyline affects spermatogenesis, and “↓ (purple/red)” indicates that mirtazapine/amitriptyline has a greater impact on spermatogenesis.

According to the plasma concentrations of amitriptyline (FDA label) and mirtazapine [9], the *in vitro* concentrations chosen by us were 100 nM and 1 μM for subsequent *in vitro* experiments. After 7 days of culture, organoids were treated with both drugs for 48 and 96 h and then stained with Hoechst (live cells) and propidium iodide (dead cells). Figure 1C and Supplementary Figures S2,S3 show that testicular organoids treated with amitriptyline contained significantly more apoptotic cells than those treated with mirtazapine, while mirtazapine treatment resulted in significantly more live cells than treatment with amitriptyline, indicating that the reproductive toxicity of amitriptyline was significantly higher than that of mirtazapine. We further quantified these effects by measuring and calculating the ratio of the fluorescence intensity of PI and Hoechst with a confocal microscope (Olympus, Tokyo, Japan). We measured the red fluorescence value represented by PI and the blue fluorescence value represented by Hoechst. After that, the ratio of red fluorescence to blue fluorescence was calculated, which indicated that the higher the R level was, the higher the cell death rate. The results showed that the R value of amitriptyline was higher than that of mirtazapine ( Figure 1D). This result was consistent with the IC _50_ data calculated in GC1-spg/GC2-spd/TM3 Leydig/TM4 Sertoli cultured mouse cell lines ( Supplementary Figure S4). Amitriptyline was significantly more cytotoxic to GC1, GC2, TM3 and TM4 cell lines than mirtazapine, which means amitriptyline would bring more damage to the testis. To determine the effects of both drugs on testicular organoids, we used RT-PCR to detect changes in markers related to germ cells after drug treatment. JQ1, a drug that blocks spermatogenesis, was used as a positive control. Stimulated by retinoic acid 8 (Stra8) and ubiquitin C-terminal hydrolase L1 (Uchl1) genes were detected ( Figure 2). When the dose of both drugs was 1 μM, the expression of Stra8 was downregulated after 48 h, suggesting that the increased dose of both amitriptyline and mirtazapine can start to block sperm maturation. However, the expression of Uchl1, a specific marker of undifferentiated spermatogonia, was increased after 48 h of treatment with amitriptyline but not with mirtazapine. This suggested that amitriptyline was more toxic than mirtazapine in the organoid culture system and that normal spermatogenesis could be affected. We then extended the administration time to 96 h, and our results showed that the expression of Stra8 was increased after treatment with 100 nM mirtazapine compared to the control, while there were no changes with amitriptyline under the same treatment. This was presumably because the two drugs damaged the undifferentiated spermatogonia (data not shown). The expression of Uchl1 was significantly downregulated at the same time, which might be due to negative feedback regulation when the expression of Stra8 was increased. We further found that the expression levels of H1.6 linker histone, cluster member (Hist1h1t) and aurora kinase C (Aurkc) were downregulated under the same treatments, which is also related to sperm maturation ( Supplementary Figure S5). These results confirmed that in this cultured organoid system, amitriptyline has much worse effects than mirtazapine, which was probably related to spermatogenesis. However, when the dose of both drugs was increased to 1 μM, the mRNA expression of Stra8 was downregulated ( Figure 2). Moreover, after the administration time was extended to 96 h, the undifferentiated spermatogonia were also damaged by the drugs and probably died (data not shown). This was reflected in the significant downregulation of Uchl1 expression ( Figure 2), indicating that the two drugs caused great damage to undifferentiated spermatogonia, which consequently destroyed the spermatogenic ability of the testicular organoid model. Subsequently, we used RT-PCR to detect changes in markers related to Sertoli cells. Zo1 and Sox9 were detected to observe the changes in the mRNA levels of Sertoli cells. Interestingly, the expression of Zo1 was increased compared to the control under treatment with both drugs (100 nM, for 48 h and 96 h, Figure 2A), suggesting that neither drug damaged the blood-testis barrier over time (data not shown). However, with the increased dose (1 μM), mirtazapine treatments of 48 h and 96 h did not change the expression of Zo1, while 96 h of treatment with amitriptyline downregulated the expression of Zo1 ( Figure 2). Under these circumstances, damage to the BTB would be caused by amitriptyline. Similarly, Sertoli cells were damaged only under treatment with amitriptyline at 1 μM for 96 h, which suggested that Sertoli cells are more resistant to these two drugs, especially mirtazapine. This result is consistent with the results from experiments on cultured cells ( Supplementary Figure S4).

The above results also suggested that the developed organoid can be used to assess the reproductive toxicity of antidepressants and uncover how the drugs affect spermatogonia. By detecting the mRNA of the organoid with the two drug treatments, we can summarize that both drugs can damage spermatogonia and primary spermatocytes in the early stage of differentiation, suggesting that both drugs may affect the proliferation and division of spermatogenic cells. In addition, in the spermatogenesis process, amitriptyline can have a more significant effect on the expressions of Sox9, Stra8, Uchl1, and Zo1 than mirtazapine, which might indicate a disruption of signalling pathways. Third, 96 h of treatment with amitriptyline could have profound effects on Sox9 and Zo1, which may indicate that the use of amitriptyline in clinical practice may cause more serious damage to the male reproductive system ( Figure 2B).

Finally, we used mouse cell lines such as GC1 and TM4 to compare the reproductive toxicity of these two drugs with the results obtained on the organoid developed in this study ( Supplementary Figure S6). After the cell lines were treated with amitriptyline and mirtazapine for 24 h, the expression levels of key genes such as *Slc6a4*, *Slc6a2*, *Htr2c*, *Oprk1*, *Hrh1*, and *Adra2a* changed dramatically ( Supplementary Figure S6). It has been reported that changes in the expression levels of the above genes could lead to impaired testosterone synthesis, arrest of spermatogenesis, and harmful effects on male fertility [10]. These results are consistent with our results on organoids in this study. However, testicular organoids contain major cell types, including Sertoli cells and germ cells of different stages and with more physiological structures and tight junctions, such as the blood testis barrier; therefore, the model can reflect the *in vivo* effects of the drugs. In addition, the testicular organoids can reflect the key genes in the sperm pathway much closer to the environment *in vivo* ( Supplementary Figure S7). Therefore, the organoid developed in this study can be used as an effective model for evaluating drug effects on sperm production *in vitro*.

In short, the organoids that we cultured for 7 days contained spermatogonia, spermatocytes and Sertoli cells, and developed a blood-testis barrier, which indicated that the organoids have the potential to mimic sperm production *in vitro*. Indeed, supporting cells and spermatocytes that are essential in the sperm production process were also present in the organoid models. Spermatogenesis has specific metabolic requirements, and the existence of the blood-testis barrier indicated that organoids could provide the regulatory microenvironment conditions required during spermatogenesis. The rat testicular organoids developed in this study have the potential to mimic sperm development and to be used to assess male reproductive toxicity.

Of note, the current study is a preliminary study which has several limitations that need further improvement. First, the “organoid model” is still in the preliminary stage, with no specific testicular cytoarchitecture/morphology. Second, the present evidence is not convincing enough to support that this model replicates testis-specific structure and function. Third, although the marker expression of spermatogonia, Sertoli cells and other cell types can be determined, whether germ cells differentiate during spermatogenesis requires further investigation. Nevertheless, the current model is suitable for drug toxicity screening and could potentially be used for the mechanistic study of drugs on spermatogenesis. Although it cannot replace *in vivo* animal studies, it can hopefully reduce the number of animals used for reproductive toxicity studies of psychological drugs. Additionally, this organoid culture method has high repeatability and easy operability, and our study shows that this organoid model can discern the effect of different drugs in this cultured organoid system. This provides a powerful tool to obtain a more comprehensive picture of the dynamic process of spermatogenesis and to analyse key pathways in specific cell types differentially affected by the drugs. This type of evaluation of reproductive toxicity in rat testicular organoids might pave the way towards developing human testicular organoid models and to provide insights into the reproductive toxicity of clinically relevant drugs in humans.
